# Simulation and retrofitting of mass exchange networks in fertilizer plants

**DOI:** 10.1038/s41598-023-40924-w

**Published:** 2023-08-24

**Authors:** Abeer M. Shoaib, Amr A. Atawia, Mohamed H. Hassanean, Ahmed A. Bhran

**Affiliations:** 1 Department of Petroleum Refining and Petrochemical Engineering, Faculty of Petroleum and Mining Engineering, Suez University, Suez, Egypt; 2 Chemical Engineering Department, College of Engineering, Imam Mohammad Ibn Saud Islamic University, Riyadh, Saudi Arabia

**Keywords:** Chemical engineering, Environmental chemistry

## Abstract

This paper presents a simulation technique for optimizing a hydrogen integration network. By applying this technique, the minimum fresh hydrogen consumption can be determined. Quantitative relationship between sources and sinks streams were studied to get the flow rates of coupled source and sink, hydrogen consumption and hydrogen concentration in each stream. The introduced technique was applied on twelve sources and twelve sinks with any purity of hydrogen concentration. The hydrogen integration network was designed through two steps, the first step considers applying the data given in the LINGO program, while the second step considers using the LINGO results in the introduced excel program to obtain the retrofitted hydrogen integration network. The proposed technique was applied on several case studies to achieve the minimum consumption of fresh hydrogen for the obtained hydrogen integrated networks. The introduced model for simulation and retrofitting of mass exchange networks is easy to understand and the results showed that this model is more efficient for fertilizer, petrochemical and refinery plants.

## Introduction

Reduction in the consumption of fresh hydrogen in hydrogen integration networks is a challenge in many plants, such as fertilizers plants, oil refineries and petrochemical plants.

In recent years, various methodologies were introduced to improve the hydrogen utilization in these plants by maximizing the amount of hydrogen recovered using the pressure constraints in the design ^1^. A graphical method is used to make analysis of hydrogen distribution in the network and minimize the consumption of hydrogen ^2^. Algebraic and other graphical techniques are also used to maximize direct reuse, minimize waste discharge and minimize fresh hydrogen consumption to get the effective strategy of cost reduction ^3^. Zhenhui et al. have introduced a graphical method based on composite curves in order to determine the minimum hydrogen demand ^4^. Denny et al. have introduced an automated procedure for determining the minimum hydrogen consumption using reuse/recycle technique; they applied their procedure to single impurity resource conservation network (RCN) ^5^. Genetic Algorithm is introduced by Khajehpour et al. in order to minimize the waste of hydrogen in refinery plants; that method is applied on Iranian refinery and achieved a considerable reduction in hydrogen consumption as well as the cost. ^6^. Waste interception network is presented to minimize the cost of hydrogen consumption by using an automated technique of resource conservation network (RCN) ^7^. Mixed integer nonlinear program (MINLP) is also used to minimize the operating and capital cost of hydrogen using plants ^8^. Optimum distribution of hydrogen networks is achieved using mathematical models aiming to minimize the operating and capital costs as well as the consumption of hydrogen, taking into consideration the constraints of pressure, purity and compressor flowrates ^9^. A Graphical technique known as MSCC is proposed to maximize the reuse/recycle in the hydrogen integration network with minimizing the consumption of fresh hydrogen and waste discharge^10^. Another graphical method based on pinch point is used to minimize the consumption of hydrogen utility in refinery plants^11^. Han et al. introduced a mathematical model for hydrogen supply networks^12^; they approved that the physical form of stored hydrogen is affecting the net profit of the plant. Two systematic mathematical methods based on two-step approach are introduced by Jiao et al. to increase the efficient use of hydrogen utility and retrofit the hydrogen network in refinery^13^. Almansoori and Shah introduced a multiperiod MILP model to overcome the challenges facing the design a hydrogen supply chain (HSC)^14^. Liu et al. studied the purification reuse/recycle strategy for maximizing the hydrogen saving in plants; they introduced a graphical based method to identify the pinch point hydrogen utility saving from a given purification feed flow rate^15^. LINGO The Modeling Language and Optimizer is a manual book that helping the programmers to apply their mass balance equations, assumptions and constraints to get the optimum objective function^16^. Guilian et al. handled the hydrogen networks with purification; they analyzed systematically the effect of purification feed purities and purification product purities in the hydrogen network to get the pinch point and the minimum consumption of hydrogen utility ^17^. Two different optimization techniques are also presented by Fatma et al. to maximize the hydrogen recovery in networks; they applied their techniques to Medor Refinery Plant where the consumption of hydrogen utilities and discharge have been considerably reduced ^18^. Moreover, a mathematical programming model is used to design the hydrogen network by using a purity unit to minimize the total annual cost and increase the ratio of hydrogen recovery in refineries and fertilizer plants ^19^. A purification technique by using LaNi_5_ is introduced by Kaiyu et al. to maximize the saving of hydrogen utility in the design of reuse and recycle hydrogen networks; they applied their technique to two refineries and achieved a noticeable reduction in the consumption of hydrogen utility in two different studied refinery plants^20^. Three linear programming are introduced by Qiao et al. to decrease the emission of carbon and conservation of resources in the hybrid hydrogen network for refinery and synthetic plant of chemicals; they achieved a great progress in the design of hydrogen integration network for reducing the consumption of hydrogen and carbon emission ^21^.

According to high cost of fresh hydrogen, an automated technique which solve the critical problem in the huge consumption of fresh hydrogen was designed in the present work. Reaching to the optimum distribution flow rates between sources and sinks are obtained after using LINGO software version 14.0. The structure of hydrogen integration network is based on a mathematical programming that contains equations of mass balance and component mass balance between sources and sinks with adding constrains and assumptions. That software has the ability to solve several linear and nonlinear equations quickly with availability to change the structure of any constrains, assumptions and decision variables in our mathematical approach. The objective of this work is to minimize the consumption of fresh hydrogen utility with designing an optimum hydrogen integration network. This target is achieved and processed in two programs; first program is solved by LINGO software which has the ability to solve several equations that constructed by our mathematical approach and the second program is solved by Excel software which is designed to catch the results from first program and drawing our hydrogen integration networks. Lingo optimization software version 14.0 used in this research was introduced to get the objective function of minimizing the fresh hydrogen consumption. With reusing the hydrogen streams between the processes, the consumption of fresh hydrogen will be decreased. Multi component system of pure hydrogen, impure hydrogen and component C are presented in the proposed program. Allowing the transportation of hydrogen streams between the processes is effective in improving the utilization efficiency of hydrogen. Three case studies are investigated in this study; their data are compared with the obtained results of the introduced approach. The presented case studies achieved a reduction in fresh hydrogen consumption by 5%, 3.1% and 0.25% respectively, while the discharged flowrate of hydrogen has been decreased by 10%, 7.6% and 0.75% respectively. The simulation approach is designed in two steps, the first step is to get the minimum consumption of fresh hydrogen by applying a mathematical approach and the second step is to obtain the drawing of the optimum hydrogen integration network by using the results of the simultaneous optimization program. The mathematical approach is based on mass balance equations between twelve sources and twelve sinks to get a minimum fresh hydrogen consumption.

## Methods

The objective of the current work is to design the hydrogen integration networks for the investigated case studies in the presence of multi contaminant system with using fresh hydrogen source.

Our optimization methods are formulated as Nonlinear Program (NLP) that based on overall mass balance and component mass balance equations between sources and sinks. The variables are a positive real number or zero values because the variables referred to the flowrate from sources to sinks, flowrate of hydrogen utility, flowrate of waste, the inlet concentrations of sinks, out let concentrations of sources and the concentrations of waste discharge.

The problem definition can be stated as follows: Given a set of sources, each source (n) has a flow rate (F_Sn_) and concentration of multi contaminant (X_SnA_, X_SnB,_ X_SnC_), Where contaminant A refer to the impurity of hydrogen, Contaminant B refer to purity of hydrogen and contaminant C refer to a component in the production cycle such as nitrogen or oxygen or sulfur dioxide or carbon dioxide, these contaminants can be reused or recycled or discharged.

Given a set of sinks, each sink (m) has a flow rate (G_m_) and limiting concentration of multi contaminant (Z_mAin_, Z_mBin,_ Z_mCin_) that is lower than the maximum allowable concentration. The objective function of this research work is the minimization of the fresh hydrogen consumption. This can be achieved by retrofitting the investigated hydrogen network using the LINGO optimization software and then the LINGO results are used to feed the introduced program to get the final hydrogen integration network.

**Figure 1:**
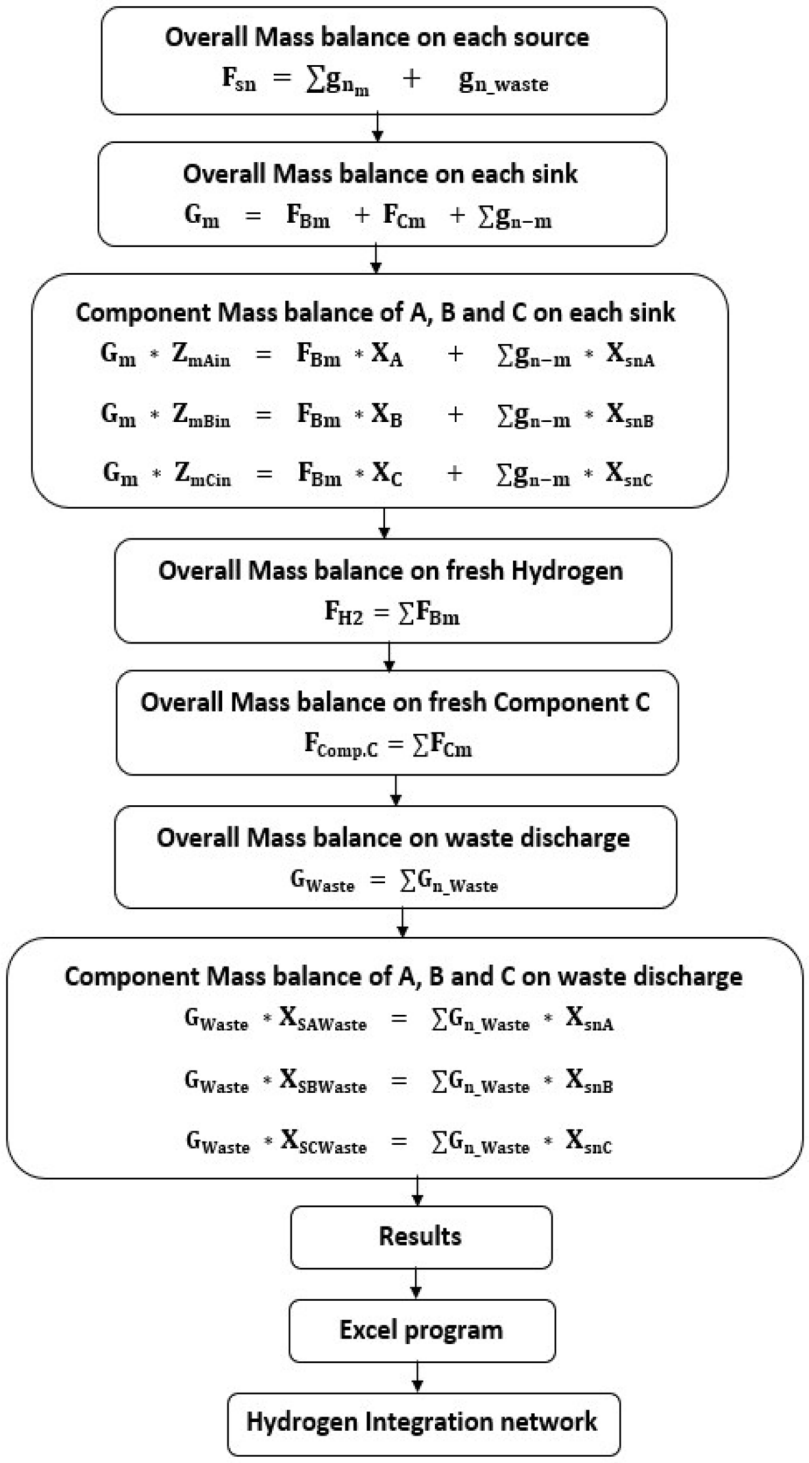
Procedure used for designing the hydrogen integration network.

Figure 1 illustrates the procedure used for designing the studied hydrogen network which started by an overall mass balance which applied on each source (n), the flow rate of source (F_sn_) distributed to both ways, first way is to sinks by flow rate (${\text{g}}_{{{\text{n}}_{{\text{m}}} }}$) and the second way is to waste by flow rate (${\text{g}}_{{{\text{n}}_{{{\text{waste}}}} }} )$ as shown in Eq. (1).

1 $$ {\text{F}}_{{{\text{sn}}}} = \sum {\text{g}}_{{{\text{n}}_{{\text{m}}} }} + {\text{ g}}_{{{\text{n}}\_{\text{waste}}}} $$

As shown in Eq. (2), we applied overall mass balance on each sink (m) where the flow rate of each sink (${\text{G}}_{{\text{m}}}$) represent the summation of flow rate of fresh hydrogen (${\text{F}}_{{{\text{Bm}}}}$), flow rate of fresh component C (${\text{F}}_{{{\text{Cm}}}}$) and the flow rate of summation flow rates from sources to sinks ($\sum {\text{g}}_{{{\text{n}} - {\text{m}}}}$).

2 $$ {\text{G}}_{{\text{m}}} { } = {\text{ F}}_{{{\text{Bm}}}} { } + {\text{ F}}_{{{\text{Cm}}}} { } + { }\sum {\text{g}}_{{{\text{n}} - {\text{m}}}} $$

A component mass balance applied at each sink on three components, component A (impurity of hydrogen), component B (Purity of hydrogen) and component C, as it is mentioned before component C may be nitrogen or oxygen or sulfur dioxide or carbon dioxide. As shown in Eqs. (3, 4 and 5), there are different concentrations (Z_mAin_, Z_mBin_ and Z_mCin_) of components A, B and C respectively entered to sink (m) and the concentrations (X_A_, X_B_, X_C_) represent the impurity of hydrogen, purity of hydrogen and concentration of component C respectively, where the concentrations (${\text{X}}_{{{\text{snA}}}} , {\text{X}}_{{{\text{snB}}}}$ and ${\text{X}}_{{{\text{snC}}}}$) represent the concentration of components A, B and C respectively entered from source (n) to sink (m).

3 $$ {\text{G}}_{{\text{m}}} * {\text{ Z}}_{{{\text{mAin}}}} { } = {\text{ F}}_{{{\text{Bm}}}} {\text{ *X}}_{{\text{A}}} + { }\sum {\text{g}}_{{{\text{n}} - {\text{m}}}}  *{\text{ X}}_{{{\text{snA}}}} $$

4 $$ {\text{G}}_{{\text{m}}}  * {\text{Z}}_{{{\text{mBin}}}} { } = {\text{ F}}_{{{\text{Bm}}}} {\text{ *X}}_{{\text{B}}} { } + { }\sum {\text{g}}_{{{\text{n}} - {\text{m}}}}  * {\text{X}}_{{{\text{snB}}}} $$

5 $$ {\text{G}}_{{\text{m}}} * {\text{ Z}}_{{{\text{mCin}}}} { } = {\text{ F}}_{{{\text{Bm}}}}  *{\text{X}}_{{\text{C}}} { } + { }\sum {\text{g}}_{{{\text{n}} - {\text{m}}}}  * {\text{X}}_{{{\text{snC}}}} $$

Also, an overall mass balance was applied on total fresh hydrogen consumption, fresh component C and waste discharge stream (G_waste_) as shown in Eqs. (6, 7 and 8).

6 $$ {\text{F}}_{{{\text{H}}2}} = \sum {\text{F}}_{{{\text{Bm}}}} $$

7 $$ {\text{F}}_{{{\text{Comp}}.{\text{C}}}} = \sum {\text{F}}_{{{\text{Cm}}}} $$

8 $$ {\text{G}}_{{{\text{Waste}}}} { } = { }\sum {\text{G}}_{{{\text{n}}\_{\text{Waste}}}} $$

Furthermore, a component mass balance was applied on components A, B and C on the discharge stream as shown in Eqs. (9, 10 and 11).

9 $$ {\text{G}}_{{{\text{Waste}}}} {\text{ *X}}_{{\text{SAWaste }}} { } = { }\sum {\text{G}}_{{{\text{n}}\_{\text{Waste}}}}  * {\text{X}}_{{{\text{snA}}}} $$

10 $$ {\text{G}}_{{{\text{Waste}}}} {\text{ *X}}_{{\text{SBWaste }}} { } = { }\sum {\text{G}}_{{{\text{n}}\_{\text{Waste}}}}  * {\text{X}}_{{{\text{snB}}}} $$

11 $$ {\text{G}}_{{{\text{Waste}}}} {\text{ *X}}_{{\text{SCWaste }}} { } = { }\sum {\text{G}}_{{{\text{n}}\_{\text{Waste}}}} * {\text{ X}}_{{{\text{snC}}}} { } $$

Adding a constrains (All flow rates and concentrations have a positive value or zero) is recommended in our mathematical model to get the optimum solution.

After running our mathematical approach in LINGO optimization software, we get the minimum consumption of fresh hydrogen flowrate with determining each flow rate from sources to sinks (g_n-m_), fresh hydrogen consumed in each sink (F_Bm_), fresh component C consumed in each sink (F_Cm_), total fresh component C (F_comp.C_), waste flow rate discharges from each source (g_n-waste_), total waste discharge from all sources (G_waste_), as well as concentrations of component A, B and C in the discharge waste (X_SAWaste_, X_SBWaste_, X_SCWaste_).

**Figure 2:**
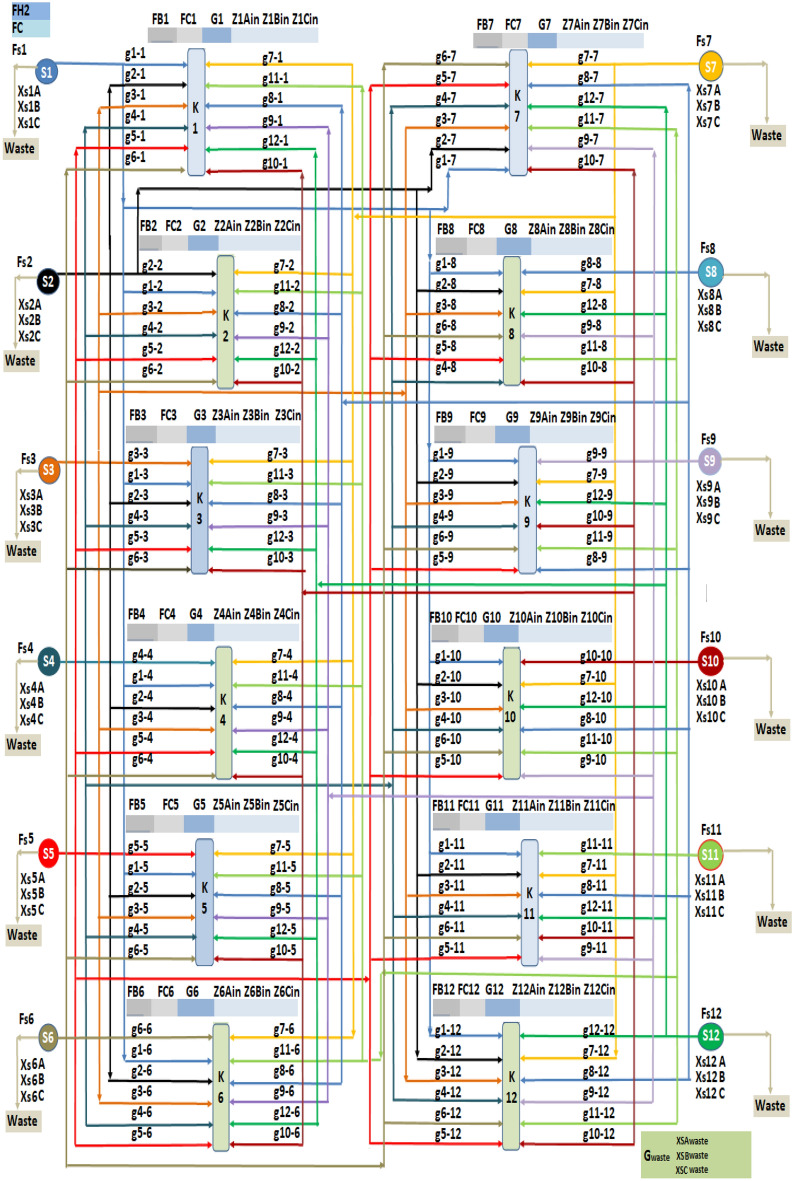
Allocation of sources and sinks in investigated hydrogen integration network case studies.

By applying the results from LINGO optimization software and the introduced Excel software on the investigated case studies, the drawing of the hydrogen integration network is obtained automatically which shows the minimum fresh hydrogen consumption and all flow rates from sources to sinks with concentrations and all discharge flow rates from sources with their concentrations. As shown in Fig. 2, twelve sources distribute their flow rate streams to the sinks units. In each sink, the flow rates of fresh hydrogen (F_Bm_) and fresh component C (F_Cm_) with twelve streams from the twelve sources (g_n-m_), the concentration of each sink (Z_mAin_, Z_mBin_, Z_mCin_) appears up on each sink drawing. Additionally, the minimum fresh hydrogen consumption (F_H2_) and minimum fresh component C consumption (FC) are shown at the top of the obtained hydrogen integration network. The total discharge flow rate (G_waste_) and the concentrations of their contaminates (X_SAWaste_, X_SBWaste_, X_SCWaste_) are presented at the bottom of the obtained hydrogen integration network. The proposed method is easy to use and understand, after we adding the data given of flowrate of sources and sinks with their concentrations the mathematical model will run and the results will send to the excel software to draw the hydrogen integration network automatically. Our optimization technique program has the ability to design a network between a number of sources and sinks reach to twelve source and twelve sink with a different purity of hydrogen utility.

## Case studies

The introduced retrofitting technique in the present research work was tested to show its effectiveness by applying this technique on three case studies as described in the following subsections.

### Case study 1

The first case study of this work was described by Chun Deng et al. ^18^, for a fertilizer plant. This plant includes six sources coming from the catalytic reforming unit (CRU), hydrocracker unit (CHU), gas oil hydrotreater (GOHT), residue hydrotreater (RHT), diesel hydrotreater (DHT) and naphtha hydrotreater (NHT). The five sinks are coming from the hydrocracker, gas oil hydrotreater, residue hydrotreater, and diesel hydrotreater and naphtha hydrotreater units with using 89,304 Nm^3^/h of currently hydrogen utility with a purity of 95%, flow rates and hydrogen purity of sources and sinks represented in Table 1.

**Table1:** Flow rates and hydrogen purities of sources and Sinks streams for case study 1.

Sources and sinks	Stream number	Units	Flow rate (Nm^3^/h)	H_2_ purity (mole fraction)
Sources	1	CRU	17,303	0.8
	2	HCU	60,678	0.8
	3	GOHT	55,281	0.75
	4	RHT	25,870	0.75
	5	DHT	8004	0.7
	6	NHT	3840	0.65
Sinks	1	HCU	93,306	0.8671
	2	GOHT	82,656	0.8358
	3	RHT	39,164	0.8257
	4	DHT	12,472	0.7487
	5	NHT	5726	0.7265

### Case study 2

The second case study of the current work is the ethylene plant investigated by Chun Deng et al. ^18^. This plant six sources are the steam reformer unit (SRU), catalytic reforming unit, hydrocracker unit, naphtha hydrotreater, diesel hydrotreater and cracked naphtha hydrotreater (CNHT). The four sinks of this plant are the hydrocracker unit, naphtha hydrotreater, diesel hydrotreater and cracked naphtha hydrotreater. The current hydrogen utility is 22,353 Nm^3^/h with hydrogen purity of 95%, flow rates and hydrogen purities of sources and sinks are addressed in Table 2.

**Table 2:** The limiting data of sources streams for Case study 2.

Sources and sinks	Stream number	Units	Flow rate (Nm^3^/h)	H_2_ purity (mole fraction)
Sources	1	SRU	50,303	0.93
	2	CRU	33,530	0.8
	3	HCU	145,305	0.75
	4	NHT	11,177	0.75
	5	DHT	27,942	0.73
	6	CNHT	36,885	0.7
Sinks	1	HCU	201,197	0.8061
	2	NHT	14,531	0.7885
	3	DHT	44,707	0.7757
	4	CNHT	58,117	0.7514

### Case study 3

The third case study was taken from the research work done by Shoaib et al.^17^. This work considers Medor refinery. This plant has nine hydrogen sources come from the naphtha hydrotreater, isomerization (ISO), diesel hydrotreater, hydrocracking (HC), pressure swing adsorption (PSA), and catalytic reforming units. However, the six sinks of this plant come from naphtha hydrotreater, isomerization, diesel hydrotreater, Hydrocracking, and pressure swing adsorption units. The current hydrogen utility is 2263.67 Kgmole/h with a purity of 99.99%. The data of flow rates and purities of sources and sinks are shown in Table 3.

**Table 3:** The limiting data of sources streams for Case study 3.

Sources and sinks	Stream number	Units	Flow rate (kgmole/h)	H_2_ purity (mole %)	H_2_ impurity (mole %)
Sources	1	NHT	20.25	95.62	4.38
	2	NHT	97.9	46.84	53.16
	3	ISO	122.6	41.78	58.22
	4	DHT	2396.94	83.04	16.96
	5	HC	22,434.72	89.21	10.79
	6	HC	515.15	73.48	26.52
	7	PSA	1011.19	99.9	0.1
	8	PSA	276.61	54.57	45.43
	9	CRU	2141.2	90.186	9.814
Sinks	1	NHT	2024.99	95.62	4.38
	2	NHT	135.56	90.22	9.78
	3	ISO	241.3	90.22	9.78
	4	DHT	2873.48	84.23	15.77
	5	HC	3276.9	99.9	0.1
	6	PSA	22,434.72	89.21	10.79

## Results and discussions

By entering the data given in our mathematical approach in LINGO optimization software and run the program, the obtained results show the flowrates from sources to sinks, flowrate of fresh hydrogen to each sink, flow rate of each discharge stream and the concentrations of the components in each stream. The introduced Excel software uses the results from LINGO optimization software and the drawing of the hydrogen integration network is automatically achieved. The introduced approach for optimizing and retrofitting of mass exchange networks in fertilizers, ethylene and refinery plants was applied on three case studies and the results are discussed in the following subsections.

### Case study 1

After applying the LINGO optimization software for the first case study, the obtained results of streams flow rates from sources to sinks (g_n−m_) are listed in Table 4.

**Table 4:** Flow rate of sources to sinks and waste for case study 1.

Stream	Flowrate (Nm^3^/h)	Stream	Flowrate (Nm^3^/h)
g_1-1_	8814.8	g_4-2_	8755.5
g_1-2_	8488.2	g_4-5_	4380.4
g_2-1_	38,260	g_6-4_	162.1
g_2-2_	22,418	g_6-5_	1345.6
g_3-1_	3369.2	g_4-waste_	12,734.2
g_3-2_	15,261.5	g_5-waste_	8004
g_3-3_	24,340.4	g_6-waste_	2332.3
g_3-4_	12,309.9		

**Figure 3:**
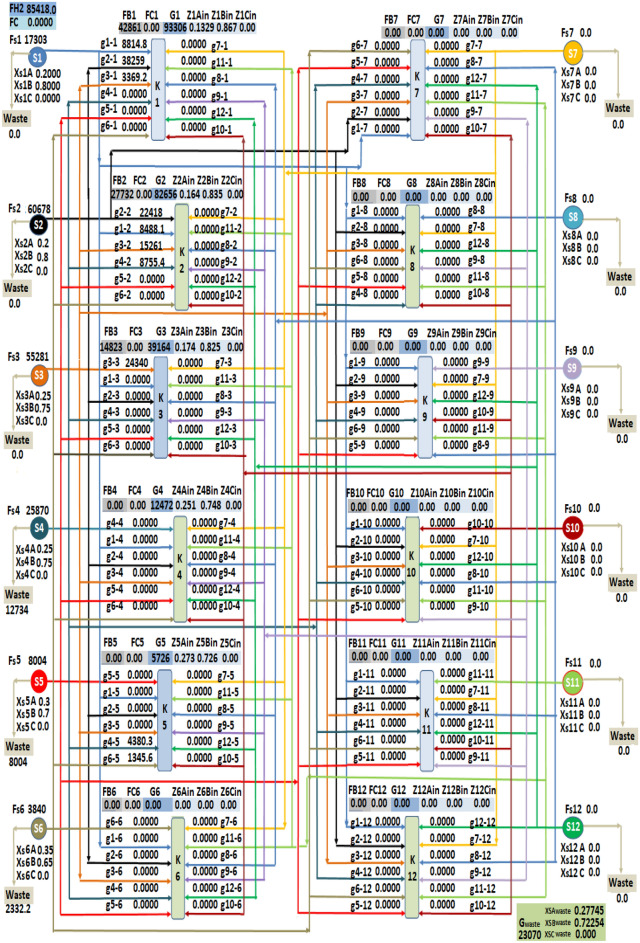
Hydrogen Integration network for case study 1.

The obtained flowrates of fresh hydrogen in sink 1, sink 2, and sink 3 are 42,861.96, 27,732.88, and 14,823.57 Nm^3^/h respectively. The results also showed that the fresh hydrogen is free of component C and the total flowrate of fresh hydrogen consumption is 85,418.41 Nm^3^/h. The achieved discharge (G_waste_) flowrate is 23,070.41 Nm^3^/h and the concentration of component A, and component B in mole fraction for this discharge are 0.2774562, and 0.7225438 respectively. Figure 3 shown the final hydrogen integration network after the results sent to the excel software.

By comparing the obtained results with the data of the original plant, it is noticed that the consumption of fresh hydrogen decreased from 89,304 to 85,418.41 Nm^3^/h and the discharge flow rate decreased from 25,635 to 23,070.41 Nm^3^/h, This consequently shows that the application of the mathematical approach leads to decrease the consumption of fresh hydrogen and the discharge flow rate by 5% and 10% respectively.

### Case study 2

**Figure 4:**
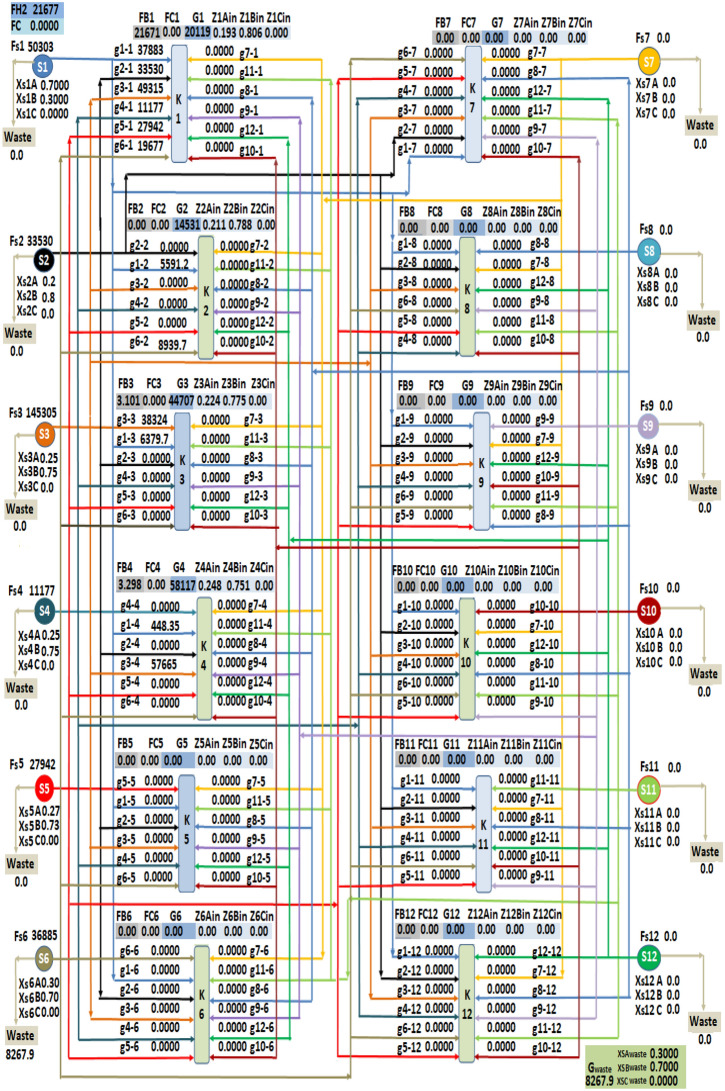
Hydrogen Integration network for case study 2.

The second case study investigated in the present work is the ethylene plant. The data given of this plant are applied on the introduced mathematical approach (LINGO optimization software), then the obtained results are used for the excel software to get the final draw of the optimum hydrogen integration network. The flow rates from sources to sinks are shown in Table 5 and the hydrogen integration network is presented in Fig. 4.

**Table 5:** Flow rate of sources to sinks and waste for case study 2.

Source	Flowrate (Nm^3^/h)	Sinks				Waste
		K1	K2	K3	K4	0
S1	50,303	37,883.65	5591.28	6379.72	448.36	0
S2	33,530	33,530	0	0	0	0
S3	145,305	49,315.48	0	38,324.18	57,665.35	0
S4	11,177	11,177	0	0	0	0
S5	27,942	27,942	0	0	0	0
S6	36,885	19,677.36	8939.72	0	0	8267.92

The obtained results show that the total consumption of fresh hydrogen flowrate is 21,677.92 Nm^3^/h which is distributed to sink 1, sink 3 and sink 4 with flowrates of 21,671.52, 3.101747, and 3.298759 Nm^3^/h respectively. The discharge flowrate is 8267.92 Nm^3^/h with concentrations in mole fraction of 0.3, 0.7 and 0 for components A, B and C respectively. The consumption of fresh hydrogen decreases from 22,353 to 21,678 Nm^3^/h by a percentage of 3.1% and the discharge flow rate decreases from 8943 to 8267.916 Nm^3^/h by a percentage of 7.6%.

### Case study 3

**Figure 5:**
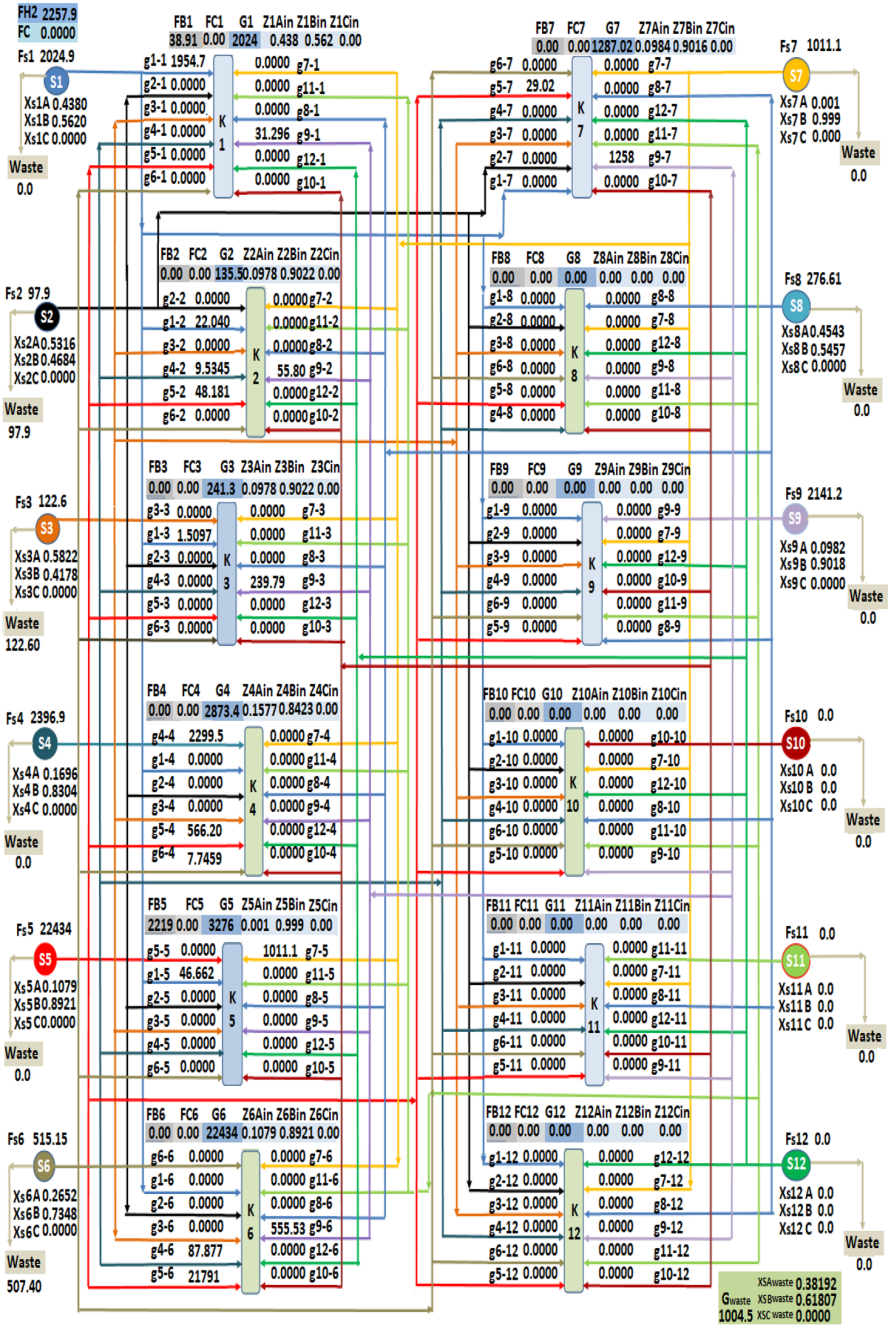
Hydrogen Integration network for case study 3.

The data given of the third case study of MEDOR plant is addressed in Table 3. The achieved results of sources flow rates to sinks after applying this plant data in the introduced mathematical model are shown in Table 6. These results are sent to the excel software to obtain the final hydrogen integration network draw shown in Fig. 5.

**Table 6:** Flow rate of sources to sinks and waste for case study 3.

Sinks	Sources flow rates (kgmole/h)								
	S1	S2	S3	S4	S5	S6	S7	S8	S9
K1	1954.77	0	0	0	0	0	0	0	31.29
K2	22.04	0	0	9.53	48.18	0	0	0	55.80
K3	1.51	0	0	0	0	0	0	0	239.79
K4	0	0	0	2299.53	566.21	7.75	0	0	0
K5	46.66	0	0	0	0	0	1011.19	0	0
K6	0	0	0	87.88	21,791.30	0	0	0	555.54
K7	0	0	0	0	29.03	0	0	0	1258.77
waste	0	97.9	122.6	0	0	507.40	0	276.61	0

Regarding the results, decreasing of the total consumption of fresh hydrogen flow rate from 2263.67 to 2257.9641 kgmole/h and decreasing of the discharged flow rate from 1012.26 to 1004.5141 kgmole/h is noticed. It is also observed that the distribution of fresh hydrogen flow rate feeds to sink 1 and sink 5 by 38.91624 and 2219.048 Kgmole/h respectively. The concentration of three components A, B and C of discharged flow rates are 0.3819243, 0.6180757 and zero respectively.

The obtained flowrates of fresh hydrogen in sink 1 and sink 5 are 38.91624 and 2219.048 kgmole/h respectively. The obtained fresh hydrogen is free of component C and the total flowrate of fresh hydrogen consumption reduced from 2263.67 to 2257.964 kgmole/h by a reduction percentage 0.25%. The achieved discharge (G_waste_) flowrate is decreased from 1012.26 to 1004.51 kgmole/h by a reduction percentage 0.75% and the concentration of component A, and component B in mole fraction for this discharge are 0.3819243 and 0.6180757 respectively.

## Conclusion

An optimization Software technique was proposed in this research to minimize the consumption of fresh hydrogen in fertilizers and refineries plants. The results produced from the simulating program is used by the proposed excel software. Drawing of the final hydrogen integration network is obtained automatically from the excel software. In the present work, the hydrogen integration network is supplied by two fresh sources, one of them is fresh hydrogen source while the other one is fresh component C. LINGO optimization software used to design a network between twelve streams of sources and twelve streams of sinks with applying overall material balance and component mass balance equations between sources and sinks. Two mathematical models are introduced in this paper to optimize the hydrogen integration network with obtaining the minimum consumption of fresh hydrogen. Respectively, the data given are applied on the optimization program and the results are entered to the excel software to obtain the hydrogen integration network. In the proposed approach, several case studies of fertilizers, ethylene and refinery plants are presented. The results show that the introduced software can be applied effectively for the investigated industrial case studies. The optimization results show that the obtained amount of fresh hydrogen consumption is reduced compared to its value for all the investigated case studies.

## Data Availability

All data generated or analyzed during this study are included in this published article.
